# Editorial: Vascular and perivascular contributions to neurodegeneration

**DOI:** 10.3389/fnins.2023.1290102

**Published:** 2023-09-29

**Authors:** Giuseppe Barisano, Daniel Bos, Yuan Ma

**Affiliations:** ^1^Department of Neurosurgery, Stanford Medicine, Stanford University, Stanford, CA, United States; ^2^Department of Radiology and Nuclear Medicine, Erasmus MC, Rotterdam, Netherlands; ^3^Department of Epidemiology, Erasmus MC, Rotterdam, Netherlands; ^4^Department of Epidemiology, Harvard T. H. Chan School of Public Health, Harvard University, Cambridge, MA, United States

**Keywords:** perivascular space, blood-brain barrier, neurodegeneration, cerebral vasculature, biomarker, glymphatic system, intramural periarterial drainage pathway, paravascular space

The cerebral vasculature, with its extensive network of arteries, veins, capillaries, and perivascular compartments, constitutes a substantial component of the brain and provides indispensable support to brain cells. In fact, the proper functioning of neurons and neuronal circuits is strongly dependent on continuous and sufficient oxygen and glucose supply from the blood, and in general, a healthy brain relies on healthy cerebral blood vessels. The cerebral vasculature also presents unique properties, especially the blood-brain barrier (BBB), that prevent solutes and cells in the blood from non-selectively crossing into the extracellular fluid and the parenchyma of the central nervous system where neurons and other brain cells reside. Moreover, recent preclinical data support a critical role of the paravascular and the intramural periarterial compartments as drainage systems of the brain, while clinical studies investigate MRI-visible perivascular spaces (PVS) in the context of neurodegeneration. These findings have significant implications for a better understanding of the pathogenesis and development of therapeutic approaches in several neurological disorders, especially neurodegenerative diseases.

The purpose of this Research Topic was to increase knowledge of the role played by the cerebral vasculature in neurodegenerative diseases, such as cerebral small vessel disease, vascular cognitive impairment, Alzheimer's disease (AD), and dementia. In recent years, novel neuroimaging methods and technological advancements enabled improvements in the visualization and quantification of the cerebral vasculature and its multiple components, including PVS and BBB. From a clinical point of view, these advancements are important for optimizing current diagnostic frameworks, identifying early biomarkers, and developing novel therapeutic strategies for neurodegenerative disease utilizing the cerebral vasculature.

In their review article, Lynch et al. give an overview of the current knowledge and gaps in understanding the contribution of PVS to AD. In particular, after describing AD, established AD biomarkers, and the glymphatic system, they summarized results from studies investigating the potential use of MRI-visible PVS as a non-invasive imaging biomarker in AD as well as the relationship between MRI-visible PVS, amyloid-beta, and tau. The review highlighted the need for further studies investigating these relationships, both cross-sectionally and longitudinally, to improve our comprehension of the role of PVS in AD in humans.

Another review article by Pham et al. summarized the recent developments in PVS segmentation and quantification on MRI. They described the current approaches for evaluating PVS in humans, including visual rating scales, segmentation, and machine learning techniques, and made comparisons between these techniques when feasible. This review also summarized the current challenges in PVS segmentation and proposed potential solutions.

In the study by Moses et al., the authors evaluated the utility of PVS burden assessed on MRI as a biomarker in patients with behavioral variant frontotemporal dementia. They used an automated technique to segment PVS on T1-weighted images. In their exploratory study with 12 patients, they found a trend toward an association between PVS burden and disease progression, as well as between the measures of tau and neurofilament light chain in the cerebrospinal fluid and PVS burden longitudinally. Their study suggests a potential involvement of PVS in neurodegenerative changes observed in these patients. Further studies are needed in order to understand the possible use of PVS as a biomarker for disease progression and/or as a therapeutic target in frontotemporal dementia.

The glymphatic system clearance activity is based on aquaporin-4 (AQP4) water channels expressed on the end-feet of astrocytes lining on the parenchymal side of the perivascular compartment. The study by Wang et al. in rats with painful diabetic neuropathy manifesting mechanical allodynia and impairment in the glymphatic system showed that β-Hydroxybutyrate is able to restore AQP4 polarity in the spinal glymphatic system leading to enhanced glymphatic clearance and reduced mechanical allodynia compared with untreated animals. These encouraging results provide a promising therapeutic strategy for diabetic neuropathy.

The BBB is a vascular component mediating the interaction between the blood, PVS, and the brain. In their review, Yuan et al. focused on brain endothelial cells of the BBB, and their role in the pathophysiology of neurodegenerative disorders. In particular, the authors described the studies investigating how active signals released and received by brain endothelial cells may contribute to the pathogenesis of neurodegeneration, both facilitating the accumulation and deposition of pathological proteins and through pathways independent of pathological proteins, such as neuroinflammation. A better understanding of the molecular mechanisms underlying BBB and brain endothelial cell dysfunction will be critical for the development of therapeutic strategies targeting the blood vessels.

Another important component of the brain vasculature is represented by the vascular smooth muscle cells, which play a key role in cerebrovascular dynamics, enabling the appropriate provision of oxygen and nutrients to the brain. In their review, Hayes et al. described the role of vascular smooth muscle cells in the most common neurodegenerative disorders and what biological signaling pathways and physiological systems may contribute to their dysfunction. Moreover, they reviewed recent studies investigating cerebrovascular reactivity dysfunction and cerebral blood flow in patients with neurodegenerative and/or cerebrovascular pathologies and discussed potential therapeutic opportunities for these disorders specifically targeting vascular smooth muscle cell dysfunction.

In summary, these papers have collectively improved our understanding of perivascular and vascular contributions in Alzheimer's disease and other neurodegenerative conditions. These outcomes establish a basis for future studies and new opportunities for developing imaging biomarkers and interventional strategies for neurodegenerative disorders.

## Author contributions

GB: Writing—original draft, Writing—review and editing. DB: Writing—review and editing. YM: Writing—review and editing.

